# Why has canine rabies remained endemic in the Kilosa district of Tanzania? Lessons learnt and the way forward

**DOI:** 10.1186/s40249-015-0085-6

**Published:** 2015-11-30

**Authors:** M. J. Kipanyula

**Affiliations:** Department of Veterinary Anatomy, Faculty of Veterinary Medicine, Sokoine University of Agriculture, P. O. Box 3016, Chuo Kikuu, Morogoro, Tanzania

**Keywords:** Rabies, Zoonotic disease, Dog bite, Vaccination, Kilosa, Tanzania

## Abstract

**Background:**

Domestic dogs are the main targets for rabies control as they are the principal reservoir for transmission of the rabies virus to humans and other domestic animals. The purpose of this study was to identify the factors that contribute to the rabies virus infecting the human population in a rural community of Eastern Tanzania.

**Methods:**

Using a cross-sectional study design, field visits were conducted to gather information on villagers’ knowledge on and practices associated with canine rabies control and dog vaccination campaigns.

**Results:**

A total of 248 individuals were interviewed in the Kilosa district, Tanzania. Almost two-thirds (61.3 %) had a primary school education. The majority (91.1 %) of the respondents were aware that rabies is acquired through dog bites and 66.9 % knew about the clinical signs of rabies in an animal. Very few (17.7 %), however, were aware of the clinical signs of rabies in humans. Only 20.4 % of the respondents knew how rabies is controlled in dogs and 71 % were not aware of dog vaccination campaigns. The average number of dogs kept per household was 4 ± 3.3; 70.0 % of the respondents had one to five dogs, 28.3 % had six to dog dogs, and 1.6 % had 16–20 dogs. The dogs were primarily used to guard livestock and property, and to hunt. About 59.7 % of the respondents indicated that rabies was a public health problem. Low vaccination coverage was observed in the study area, with previous mass vaccination campaigns covering only 24.4 % of the dog population. Dogs appeared to have limited value in the studied community. Furthermore, there were no proper waste disposal facilities and oftentimes wild canids and felids visited the villages to scavenge on kitchen leftovers.

**Conclusion:**

Although communities in the Kilosa district had knowledge on rabies in dogs, they were not aware of the public health implication of the disease, which thus led a poor response during mass dog vaccination campaigns. Establishment of a well-coordinated rabies control program, strategic public health awareness campaigns, and active and passive surveillance systems for humans and domestic and wild animals should be considered as strategies to control and eradicate rabies.

**Electronic supplementary material:**

The online version of this article (doi:10.1186/s40249-015-0085-6) contains supplementary material, which is available to authorized users.

## Multilingual abstracts

Please see Additional file 1 for translations of the abstract into the six official working languages of the United Nations.

## Background

Rabies is an acute progressive encephalitis caused by a highly neurotropic virus in the genus *Lyssavirus* in the order *Mononegavirales*, in the family *Rhabdoviridae* [1]. Transmission to humans occurs via animal bites, mostlyby domestic dogs [2]. In developing countries, rabies continues to kill up to 70,000 people annually [3]. In Tanzania about 2,000 human deaths occur annually. Around 40 % of those who get bitten by suspected rabid animals are children under the age of 15 years [4–6].

Previous studies have indicated that clinical presentation of rabies in animals varies depending on the animal, with central nervous system disturbance and behavioral changes being the most common clinical signs [7]. The incubation period of canine rabies ranges between three and eight weeks, however, it may be as long as six months [8]. In dogs there are two forms of rabies: paralytic (dumb) and furious. In humans it is difficult to differentiate rabies from other diseases that affect the central and peripheral nervous system by clinical examination [9]. The incubation period in humans typically ranges from 1 to 3 months, but may vary from less than a week to a year [10]. Both paralytic and furious forms of rabies have been reported in humans. About 30 % of patients contract the paralytic form, which is less dramatic and takes a longer course than the furious form [11]. Patients with the furious form exhibit signs of hyperactivity, excited behavior, hydrophobia, and sometimes aerophobia [11, 12].

Human deaths due rabies are almost entirely preventable through prompt delivery of post-exposure prophylaxis (PEP). It is recommended that PEP is initiated in bite victims immediately after exposure in order to prevent the rabies infection. However in Sub-Saharan Africa, facilities for PEP storage and supply are limited and bite victims are therefore forced to travel long distances to get treatment [13, 14]. Traditionally it is believed that the transmission cycle of rabies between humans and dogs can be prevented through strict dog vaccination campaigns [15, 16]. Certainly, a lack of compliance to vaccination schedules, amongst other factors, creates a unique environment for the enzootic status of the disease [17, 18]. Additionally, strengthening the reporting system, early detection of rabid animals, mapping of endemic areas, and prompt response to treatment are considered part and parcel of the control strategies required to break the transmission cycle between humans and domestic dogs.

The purpose of this study was to determine the factors responsible for high incidences of the rabies disease in a human population of a rural community in Eastern Tanzania. The present study found that although respondents in the study area had a general understanding of rabies in dogs, they were not aware of the control measures and the public health implication of the disease, which resulted in poor response and compliance during mass dog vaccination campaigns.

## Methods

### Study area

**Fig. 1:**
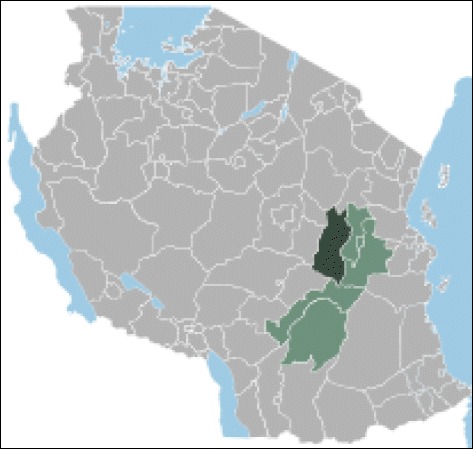
Map of the United Republic of Tanzania showing administrative regions (gray), the Morogoro region (light green), and the Kilosa district (black), where the three villages investigated in this study are located (adopted and modified from https://en.wikipedia.org/wiki/Kilosa_District)

This study was carried out in Kilosa district (5°55–7°53′S; 36°30′– 38°E), Eastern Tanzania (see Fig. 1). The district is located about 300 km from Dar es Salaam and 75 km from the town of Morogoro. The district has a total surface area of about 14,918 km^2^. According to the 2012 census, the district has an estimated population of 438,175, living in 105,635 households with an average of 4.6 people per household. The district has 38 wards, with 3,570 to 29,361 inhabitants per ward.

### Study design

This was a cross-sectional study conducted in three villages of the Kilosa district: Kiduhi, Twatwatwa, and Ihombwe. The three villages were purposively selected based on their proximity to the Mikumi National Park and due to high canine rabies incidence in the area. The choice of the villages was based on previous Kilosa District Council reports of canine and human rabies cases. The present study was a household-based study that involved face-to-face interviews with heads of households (≥18 years old). Selection of participants was based on the criterion that the household kept at least one dog.

### Data collection

The study was carried between August and September, 2013. The study participants were recruited and interviewed in their homes during a dog vaccination campaign in the area. A semi-structured questionnaire was administered in Kiswahili. The questionnaire gathered information on the number of dogs owned; the education level of the respondents; the respondents’ general knowledge about rabies, awareness of the clinical signs of rabies in animals and humans, knowledge about the control of rabies, and awareness of dog vaccination campaigns; and practices associated with the use of toilets/pit latrines and disposal of food leftovers.

### Data analysis

Data were entered in Microsoft Excel and analyzed using SPSS version 16.0. The chi-square test and the Fisher’s exact test (two-tailed) were used to evaluate statistical differences in community members’ responses. A *p*-value of less than 0.05 was considered to be statistically significant.

Permission to carry out this study was granted by District Directors of respective and ethics approval for the study was given by the Ethical Committee of Sokoine University of Agriculture (SUA), Morogoro, Tanzania.

### Ethical considerations

Permission to carry out this study was granted by the Kilosadistrict executive director. Ethics approval for the study was given by the Ethical Committee of the Sokoine University of Agriculture (SUA), Morogoro, Tanzania. The Vice-Chancellor of the SUA issued a research permit on behalf of the Tanzania Commission for Science and Technology (COSTECH) that permitted the study to be carried out. Prior to data collection, verbal consent was obtained from each study participant after the purpose and importance of the study was explained to him/her. The respondents were assured that they could withdraw from the study at any time.

## Results

### Rabies and vaccination status of dogs

According to the 2012 Kilosa District Council livestock census, there was a total of 3,574 dogs in the area (see Table 1). Twatwatwa and Malangali had the largest (905) and lowest (70) numbers of dogs, respectively. The vaccination coverage in 2012 was very low; out of the total dog population, only 871 (24.4 %) were vaccinated against rabies. Vaccination was carried out in six villages only, with coverage differing from place to place: Kimamba (96.6 %), Twatwatwa (5.5 %), Mbwade (39.4 %), Ulaya (21.2 %), Mikumi (36.0 %), and Kilosa town (69.1 %). Other villages did not vaccinate dogs at all. In 2012, a total of 30 bite cases in humans were reported: 12 in Kimamba, nine in Twatwatwa, three in Kiduhi, and six in Kilosa town. A total of 13 dogs were suspected to be rabid and were killed by villagers to minimize risks of potentially biting and infecting community members. Data on dog vaccination coverage and dog bite cases reported in 2012 in the different villages of Kilosa district are summarized in Table 1. These data were obtained from the Kilosa District Livestock Office.

**Table 1:** Dog vaccination coverage and dog bite cases reported in 2012 in the Kilosa district

Ward	Village	Human population	Number of dogs	Number of vaccinated dogs (%)	Dog bite cases reported	Suspected rabid dogs killed
Kimamba	Kimamba “A”	7249	177	171 (96.6)	12	2
	Kimamba “B”	6271	165	0 (0.0)	0	0
Rudewa	Twatwatwa^a^	2704	905	50 (5.5)	9	3
Madoto	Mbwade	1869	127	50 (39.4)	0	0
Tindiga	Malangali	4247	70	0 (0.0)	0	0
	Tindiga	5833	81	0 (0.0)	0	0
Kilangali	Kilangali	2723	131	0 (0.0)	0	0
	Kiduhi^a^	563	151	0 (0.0)	3	7
	Kivungu	4179	102	0 (0.0)	0	0
Ulaya	Ulaya Kibaoni	3673	189	40 (21.2)	0	0
	Mhenda	4138	263	0 (0.0)	0	0
Mikumi	Mikumi	15,166	333	120 (36.0)	0	0
	Ihombwe^a^	1773	116	0 (0.0)	0	0
Kilosa town	Kilosa town	34,236	637	440 (69.1)	6	1
Ruhembe	Kidogobasi	4562	227	0 (0.0)	0	0
Total		99,186	3,574	871 (24.4)	30	13

^a^Study area of the present study

### Community knowledge about and attitude related to rabies

A total of 248 respondents were interviewed, consisting of 230 (92.7 %) males and 18 (7.3 %) females. More than half (59.7 %) of the respondents indicated that rabies was a problem in the area, 31.5 % thought that rabies could also affect other livestock, 2.4 % said that wild animals were involved in the transmission of rabies to humans and domestic dogs, and 6.4 % believed that a dog bite was the only way of transmitting rabies to humans and livestock.

Regarding the education status of the respondents in the three villages, 32.3 % had informal education, 61.3 % had primary school education, and 6.4 % had secondary school education (see Table 2). Out of the total respondents in each village, 37.5, 31.0, and 28.6 % had informal education in Kiduhi, Twatwatwa, and Ihombwe, respectively. More than half of the respondents in Kiduhi (56.3 %), Twatwatwa (58.3 %), and Ihombwe (69.0 %) had primary education. The differences between the three study locations were statistically insignificant. The number of respondents with secondary education was significantly lower (*p* < 0.001) in Ihombwe (2.4 %), as compared to Kiduhi (6.2 %) and Twatwatwa (10.7 %).

**Table 2:** Respondents’ awareness of rabies in animals and humans

Variable	Level/response	Number of respondents (%)			
		Kiduhi (*N* = 80)	Twatwatwa (*N* = 84)	Ihombwe (*N* = 84)	Overall (%)
Education level	Informal	30 (37.5)	26 (31.0)	24 (28.6)	32.3
	Primary	45 (56.3)	49 (58.3)	58 (69.0)	61.3
	Secondary	5 (6.2)*	9 (10.7)*	2 (2.4)**	6.4
Knowledge about rabies	YES	69 (86.2)	77 (91.7)	80 (95.2)	91.1
	NO	11 (13.8)*	7 (8.3)*	4 (4.8)**	8.9
Awareness of clinical signs of rabies in animals	YES	45 (62.5)	56 (66.7)	60 (71.4)	66.9
	NO	35 (37.5)	28 (33.3)	24 (28.6)	33.1
Awareness of clinical signs of rabies in humans	YES	13 (16.2)	15 (17.9)	16 (19.0)	17.7
	NO	67 (83.8)	69 (82.1)	68 (81.0)	82.3
Knowledge about rabies control	YES	10 (12.5)**	21 (25)*	20 (23.8)*	20.4
	NO	70 (87.5)	63 (75)	64 (76.2)	79.6
Awareness of vaccination campaigns in the area	YES	21 (26.2)	27 (32.1)	24 (28.6)	29
	NO	59 (73.8)	57 (67.9)	60 (71.4)	71

*Not significantly different: *p* > 0. 0.05

**Significantly different: *p* < 0.05

The respondents’ awareness of rabies was ascertained by asking them whether they had ever heard about rabies or encountered a rabid dog. The majority of the respondents in the three villages had a basic understanding about rabies: 86.2 % of the respondents knew about it in Kiduhi, 91.7 % in Twatwatwa, and 95.2 % in Ihombwe. Comparing the three locations, the number of respondents unaware of rabies was significantly high (*p* < 0.01) in Kiduhi and Twatwatwa. Around two-thirds of the respondents in Kiduhi (62.5 %), Twatwatwa (66.7 %), and Ihombwe (71.4 %) were able to identify the clinical signs of rabies in a dog. But although respondents knew about the clinical signs of rabies in animals, they had little understanding about the clinical signs of rabies in humans; 83.8, 82.1, and 81.0 % of the respondents in Kiduhi, Twatwatwa, and Ihombwe, respectively, didn’t know about the clinical signs of rabies in humans. Respondents were also less knowledgeable about rabies control measures. Respondents in Kiduhi had a significantly lower knowledge (*p* < 0.001) about rabies control measures, as compared to those in Twatwatwa and Ihombwe. Less than a third of the respondents were aware of dog vaccination campaigns in their areas; only 26.2 % of respondents in Kiduhi, 32.1 % in Twatwatwa, and 28.6 % in Ihombwe were aware and participated in previous dog vaccination campaigns (see Table 2).

### Number of dogs owned by households in the study area

According to the 2012 Kilosa District Council livestock census, there was a total of 1,172 dogs in the study area: 905 in Twatwatwa, 151 in Kiduhi, and 116 in Ihombwe (see Table 1). The average number of dogs kept per household was 4 ± 3.3; 70.0 % of the respondents had one to five dogs per household, 28.3 % had six to 10 dogs, and 1.6 % had 16–20 dogs. Ninety-eight percent of the respondents also owned other domestic animals. When respondents were asked why they kept dogs, 91.6 % said they kept them for security and 9.4 % said it was for companionship.

### Dog vaccination coverage and challenges encountered

Unlike in previous vaccination campaigns, only 686 dogs were vaccinated against rabies in 2013, covering 58.5 % of the total dog population in the three villages, during the data collection for this study. Factors leading to high rabies incidence in the study area were: dogs and cats appeared to have limited value to the community and were mainly owned and managed by children; most dogs were free ranging, making it easy for them to be bitten or attacked by other dogs or wild animals; there was uncontrolled breeding of dogs; some villagers (agro-pastoralists) used dogs for hunting and as such there was increased contact between domestic dogs and wild animals; and during dry season when pastures are in short supply, pastoralists often illegally used protected areas to graze their animals, while accompanied by dogs. The major constraints against the control of rabies in dogs as identified by the respondents were inadequate supply of free rabies vaccines and poor advocacy during vaccination campaigns. Other constraints included a lack of identification to distinguish vaccinated versus unvaccinated dogs.

### Practices associated with the use of toilets/pit latrines and disposal of food leftovers

The role of a lack of good, clean toilets and disposal of food leftovers as two significant ways of attracting stray dogs and wild animals to residence premises was examined in this study. The use of toilets in Kiduhi and Twatwatwa (pastoralist communities) was significantly lower (*p* < 0.001) compared to use of toilets in Ihombwe (agro-pastoralist community). Whereas only 10 and 11.9 % of the respondents in Kiduhi and Twatwatwa used toilets, respectively, around 39.3 % of the respondents used toilets in Ihombwe (see Table 3). Three-quarters of the respondents in Kiduhi (73.8 %), Twatwatwa (72.6 %), and Ihombwe (71.4 %) disposed of food leftovers around their houses, which is likely to attract stray dogs and wild animals.

**Table 3:** Practices associated with the use of toilets/pit latrines and disposal of food leftovers

Variable	Level/response	Number of respondents (%)			
		Kiduhi (*N* = 80)	Twatwatwa (*N* = 84)	Ihombwe (*N* = 84)	Overall (%)
Use of toilets/pit latrines	YES	8 (10.0)*	10 (11.9)*	33 (39.3)**	16.1
	NO	72 (90)*	74 (88.1)*	51 (60.7)**	84.9
Throwing food leftovers around the house	YES	59 (73.8)	61 (72.6)	60 (71.4)	72.6
	NO	21 (26.2)	23 (27.4)	24 (28.6)	27.4

*Not significantly different: *p* > 0. 0.05

**Significantly different: *p* < 0.001

## Discussion

The present study explored and highlighted the key factors responsible for high rabies incidence in the Kilosa district, Eastern Tanzania. The study population owned a large number of dogs, which were primarily used to guard livestock and properties, and to hunt. Previous spatial and temporal distribution studies have shown that rabies is rampant in all regions of Tanzania, making it difficult to plan effective and localized control measures [5, 19–21]. Rabies control in dogs remains the only long-term and cost-effective means of preventing most human rabies cases [17, 18]. Certainly, public health preventive measures for the human population should be paralleled by programs for canine rabies control.

The studied community had at least some basic understanding about rabies, its clinical signs, and control and prevention measures in dogs. This could be explained by the relatively high literacy rates in the area; a large proportion of of the population in the study had at least primary school education. However, the community had a limited understanding of the public health implication of rabies, which may have contributed to reluctance in implementing control and preventive measures in dogs. Respondents in Kiduhi had a significantly lower knowledge about the control of rabies, as compared to respondents in Twatwatwa and Ihombwe. This could be attributed to the lifestyle of Kiduhi’s inhabitants; the majority are pastoralists who spent most of their lives moving from one area to another in search of pasture for their livestock.

The nature of dog ownership in the study area mirrored other Sub-Saharan African countries [18], where almost all dogs are free ranging (not confined). Although this is the case, it is noteworthy that only a small proportion of the African dog population is ownerless, therefore making most of the dog population accessible for vaccination [22–24], if necessary arrangements such as the delivery of free rabies vaccines are made. Consistent with previous studies, this study found that the proportion of dogs vaccinated against rabies during dog vaccination campaigns, which are widely advertised and free of charge, was closer to the World Health Organization (WHO) recommendations, as compared to owner charged vaccination schemes [23]. This suggests that in developing countries, comprehensive and participatory vaccination schemes accompanied by provision of free rabies vaccines are essential in order to eliminate and eradicate rabies in dogs, and should actually be made a requirement by the law. Furthermore, in the absence of a well-defined dog management and controlled-breeding system, most dogs in Africa will remain free ranging, making it easy for them to bite people and/or get bitten/attacked by other dogs or wild animals with rabies, thus sustaining the transmission cycle.

Attempts to control rabies in Kilosa, where it has remained endemic for over 50 years, have not been fruitful. The disease has continued to be a public health threat despite annual dog vaccination campaigns being conducted. Low vaccination coverage was observed as one of the factors responsible for the enzootic status of the disease in the area. For example, during the 2012 Kilosa District Council livestock department’s vaccination campaign, the coverage was less than 25 %. Similarly, low vaccination coverage rates have been reported in different parts of Tanzania, being far below the WHO-recommended target of 70 % [5, 19–21]. Such low dog vaccination coverage can be attributed to the costs of vaccination and signal a need to design multidisciplinary approaches and strategies to control the disease [14, 15, 22, 25]. Interestingly, the vaccination coverage in Kimamba “A” was very good (96.6 %) and was attributed to widespread advertising prior to the campaign (Dr. Y. Mgeni, Kilosa District Livestock officer personal communication, 2013).

Certainly, our findings, and those of others, indicate that the enzootic status of rabies in Tanzania is attributed to various factors such as: inadequately coordinated national rabies control programs; inefficient strategic public health awareness campaigns; weak active and passive surveillance systems for both domestic animals and humans; poorly coordinated mass vaccination programs of domestic dogs; and lack of prompt response for treatment of dog bite victims. Previous attempts to conduct mass vaccination campaigns in dogs suffered a number of challenges such as poor pre-vaccination communication between authorities and dog owners, and shortage of funds, especially in rural settings. In most cases, vaccinating dogs was considered not a priority as dogs and cats have limited value in the community. Dogs were mostly owned and managed by children who did not have the power to decide and allocate limited family resources to pay for the rabies vaccine.

The studied villages are located at a human-wildlife interphase where interactions between domestic dogs and wild canids and felids have been reported as common, thus suggesting the possibility of a reciprocal rabies transmission between the two populations [26]. Throwing food leftovers around houses was also common in all three villages studied. This practice attracted stray dogs and wild animals like wild canids and felids to come to the villages to scavenge on kitchen leftovers, further increasing the interaction between domestic and wild animals. Furthermore, pastoralists use makeshift toilet facilities rather latrines, which results in human excreta being improperly disposed which in turn attracts wild animals to the villages. This also promotes stray dogs and therefore sustains rabies in the area. Studies elsewhere have shown that in addition to massive dog vaccination campaigns, control of stray dogs through neutering has also been advocated to combat rabies [14, 15, 19]. Furthermore, there is a growing trend, especially during the dry season when pastures are in short supply, of pastoralists illegally using protected areas for grazing their animals while accompanied by dogs, thus increasing the chance of physical contact between domestic dogs and wildlife. Under such circumstances, a possibility of cross-species transmission of rabies cannot be ruled out.

Poor communication between veterinary and human health sectors is another challenge. Despite the fact that most people were aware of rabies, they didn’t know what to do in case their animals became infected or if a person is bitten by a suspected rabid dog. The community was not aware of where to report such cases or the required procedure to engage both veterinarians and public health workers. This necessitates a need for regular public awareness campaigns due to rabies being a constant public health threat in both rural and urban settings.

This study had several limitations. It was not possible to follow trends over a short period of time because data were collected at a single point in time, thus making it difficult to measure changes in the population. Also, convenient sampling may not have represented the entire population. Furthermore, self-reporting could have resulted in recall bias.

## Conclusion

Rabies is a threat to rural communities especially and to public health in general. The burden of rabies in Tanzania presents a picture epidemiologically similar to other Sub-Saharan African countries. Community members in Kilosa have a limited knowledge about the public health implications of canine rabies. The major factors leading to high canine rabies incidence have been highlighted in this paper. Given the tools and information available on how to prevent rabies, the establishment of a well-coordinated national wide interdisciplinary approach involving different stakeholders is the only practical and indispensable solution. Specifically, a well-coordinated rabies control program involving the free vaccination of dogs, strategic public health awareness campaigns, active and passive surveillance systems for both domestic animals and humans, and prompt response for treatment of dog bite victims should be given high priority.

## Additional file

Additional file 1:
**Multilingual abstracts in the six official working languages of the United Nations.** (PDF 525 kb)

## Abbreviations

PEP: post-exposure prophylaxis
SUA: Sokoine University of Agriculture
WHO: World Health Organization
COSTECH: Tanzania Commission for Science and Technology
