# Supplementary material for: Why has canine rabies remained endemic in the Kilosa district of Tanzania? Lessons learnt and the way forward
Source: Infect Dis Poverty. 2015 Nov 30;4:52. doi: 10.1186/s40249-015-0085-6 (PMC4668701; doi:10.1186/s40249-015-0085-6)
Supplement: Additional file 1: — Multilingual abstracts in the six official working languages of the United Nations. (PDF 525 kb) [file 40249_2015_85_MOESM1_ESM.pdf]

Translation of the abstract into the six official working languages of the United Nations

لماذا ظلّ داء الكلب مستوطنا في منطقة كيلوزا بـ تانزانيا ؟ الدروس المستفادة و سُبل المضي قدما

م. ج. كيبانيولا

الموجز

الخلفية: تُعتبر الكلاب المنزلية الهدف الرئيسيّ لعملية مكافحة داء الكلب باعتبارها الخزّان الأساسيّ المتسبب في انتقال فيروس الكلب للبشر و باقي الحيوانات المنزلية. و لقد كان الهدف من هذه الدراسة تحديد العوامل المساهمة في انتقال عدوى فيروس الكلب للبشر في المناطق الريفية من شرق تانزانيا.

الطُرُق: استخدام نموذج دراسة مستعرض شامل للقطاعات، كما أجريت زيارات ميدانية لاستقاء المعلومات حول مدى معرفة سكان القرى بعملية مكافحة داء الكلب و الممارسات المرتبطة به إلى جانب حملات تطعيم الكلاب.

النتائج: تمّ اجراء مقابلات مع 248 شخصا في منطقة كيلوزا، تانزانيا. حوالي ثلثين (61.3 بالمائة) كانوا قد تلقوا تعليما ابتدائيا في المدارس. كانت غالبية من تم استجوابهم (91.1 بالمائة) مدركين لأنّ داء الكلب ينتقل عبر عضات الكلاب و كان 66.9 بالمائة على علم بالأعراض الإكلينيكية لداء الكلب لدى الحيوان. لكن القلة القليلة فقط (17.7 بالمائة) كانت على علم الأعراض الإكلينيكية لداء الكلب لدى الإنسان. فقط 20.4 بالمائة فقط من المستجوبين كانوا على علم بكيفية التحكّم بداء الكلب لدى الكلاب و لم يكن 71 بالمائة منهم على علم بحملات تطعيم الكلاب. متوسط عدد الكلاب المُحتفظ بها في المنازل كان 3.3 ± 4، كان 70.0 بالمائة من المستجوبين يمتلكون من واحد إلى خمسة كلاب، 28.3 بالمائة منهم كانوا يمتلكون من ستة إلى عشرة كلاب، و 1.6 بالمائة منهم كانوا يمتلكون من 16 إلى 20 كلب. كانت الكلاب تُستعمل بشكل رئيسي في حراسة المواشي و الممتلكات إلى جانب استعمالها في الصيد. أشار حوالي 59.7 بالمائة من المستجوبين إلى أنّ داء الكلب عبارة عن مشكلة صحيّة عامة. تمت ملاحظة نسبة تطعيم منخفضة في مجال الدراسة، حيث شملت حملات التطعيم السابقة 24.4 بالمائة من الكلاب فقط. بدا أنّ للكلاب قيمة محدودة لدى المجموعة التي تمت دراستها. زيادة على ذلك، لم تكن هنالك منشآت ملائمة للتخلص من النفايات و في كثير من الأحيان كانت الكلبيّات و السّوريات تزور القرى لتقتات على بقايا الدجاج.

الخلاصة: على الرغم من أنّ سكان منطقة كيلوزا كانوا يتوفرون على معلومات حول داء الكلب لدى الكلاب، إلا أنهم لم يكونوا واعين بالآثار المترتبة للداء على الصحة العامة، و هو ما أدّى إلى استجابة متواضعة أثناء حملات التطعيم الجماعية الخاصّة بالكلاب. يجب اعتبار كلّ من تأسيس برنامج مكافحة محكم التنسيق لداء الكلب، و حملات توعية استراتيجية بالصحة العامة، و منظومات مراقبة ايجابية و سلبية خاصة بالبشر و الحيوانات المنزلية و غير المنزلية بمثابة استراتيجيات للتحكم في داء الكلب و استئصاله.

Translated from English version into Arabic by Mokhtar Oussama Alliouche, through

TRANSLATORS
WITHOUT BORDERS

为什么犬狂犬病仍在坦桑尼亚 Kilosa 区流行？吸取的教训和发展方向

M. J. Kipanyula

摘要

引言：家犬是狂犬病病毒传播至人类和其他家畜的主要储存宿主，因此，家犬是狂犬病防治的主要目标。本研究的目的在于确定坦桑尼亚东部一个农村社区人群感染狂犬病病毒的影响因素。

方法：采用横断面调查和实地走访的方法，收集村民对犬狂犬病防治和犬疫苗接种运动相关知识的知晓情况和行为特点等信息。

结果：在坦桑尼亚 Kilosa 区共调查 248 人。大约 2/3（61.3%）的受访者是小学文化程度。大多数受访者（91.1%）知晓被狗咬伤是狂犬病的感染途径，66.9%的受访者知道动物狂犬病的临床症状。然而，只有 17.7%的受访者知道人狂犬病的临床症状。仅 20.4%的受访者知道如何在犬中防治狂犬病，71%的受访者不知道犬疫苗接种运动。每户平均养犬数为 4±3.3。70.0%的受访者拥有 1-5 只犬，28.3%的有 6-10 只犬，1.6%的有 16-20 只犬。犬主要用于保护家畜和财产，以及捕猎。约 59.7%的受访者表示狂犬病是一个公共卫生问题。在本调查区疫苗接种覆盖率低。之前大规模疫苗接种运动仅覆盖该地区 24.4%的犬。犬在所调查社区的防控价值有限。此外，没有适当的垃圾处理设施，野生犬科和猫科动物经常进入村庄寻找厨余垃圾，均是当地狂犬病防治的挑战因素。

结论：虽然 Kilosa 区的社区居民对犬狂犬病有一定了解，但不清楚该病的公共卫生意义。因此，导致大规模犬疫苗接种运动效果不佳。将建立协调一致的狂犬病防治项目，开展战略性的公共卫生宣传活动，以及建设针对人群、家养和野生动物的主动和被动监控系统纳入控制并消除狂犬病的战略规划。

Translated from English version into Chinese by Zheng Qi, edited by Yang Pin, through

CHINESE CENTER FOR DISEASE CONTROL AND PREVENTION
NATIONAL INSTITUTE OF PARASITIC DISEASES

Pourquoi la rage canine est restée endémique dans le district de Kilosa de Tanzanie? Enseignements tirés et voie à suivre

M. J. Kipanyula

Résumé

Contexte : Les chiens domestiques sont les principales cibles de la lutte contre la rage, car ils constituent le principal réservoir pour la transmission du virus de la rage aux humains et aux autres animaux domestiques. Le but de cette étude était d'identifier les facteurs qui contribuent à l'infection par le virus de la rage de la population humaine dans une communauté rurale de la Tanzanie de l'Est.

Méthodologie : En utilisant une conception d'étude transversale, des visites sur le terrain ont été menées pour recueillir de l'information sur les connaissances des villageois et sur les pratiques associées aux campagnes de lutte contre la rage canine et de vaccination des chiens.

Résultats : Un total de 248 personnes ont été interrogées dans le district de Kilosa, en Tanzanie. Près des deux tiers (61,3 %) avaient un niveau d'éducation primaire. La majorité (91,1 %) des répondants savaient que la rage est acquise par les morsures de chiens, et 66,9 % connaissaient les signes cliniques de la rage chez un animal. Très peu (17,7 %), cependant, étaient au courant des signes cliniques de la rage chez les humains. Seulement 20,4 % des répondants savait comment la rage est contrôlée chez les chiens, et 71 % n'étaient pas au courant des campagnes de vaccination des chiens. Le nombre moyen de chiens gardés par ménage était de 4 ± 3,3; 70,0 % des répondants avaient de 1 à 5 chiens, 28,3 % avaient de 6 à 10 chiens, et 1,6 % avaient de 16 à 20 chiens. Les chiens étaient principalement utilisés pour garder le bétail et les biens et pour chasser. Environ 59,7 % des répondants ont indiqué que la rage était un problème de santé publique. On a pu constater la faible couverture vaccinale dans la zone d'étude, et les campagnes de vaccination de masse précédentes n'ont couvert que 24,4 % de la population canine. Il semble que les chiens aient peu de valeur dans la communauté étudiée. En outre, il n'y a pas d'installations appropriées d'élimination des déchets et des chiens, et des félins sauvages visitaient souvent les villages pour se nourrir des restes de cuisine.

Conclusion : Bien que les communautés du district de Kilosa connaissaient la rage chez le chien, ils n'étaient pas au courant des efforts des autorités de la santé publique pour lutter contre la maladie, entraînant ainsi un faible taux de réponse aux campagnes de vaccination de masse des chiens. La mise en place d'un programme de contrôle de la rage bien coordonné, la mise en œuvre de campagnes de sensibilisation stratégique sur la santé publique et l'établissement d'un système de surveillance active et passive pour les humains et les animaux domestiques et sauvages devraient être considérés comme des stratégies pour lutter contre la rage et l'éradiquer.

Translated from English version into French by Jean Heon, through

TRANSLATORS
WITHOUT BORDERS

Почему бешенство собак осталось эндемическим в округе Килоса Танзании? Полученный урок и перспектива на будущее

М. Дж. Кипаньяла

Реферат

Предисловие: Домашние собаки представляют собой главную цель борьбы с бешенством, поскольку они являются основным резервуаром для передачи вируса бешенства людям и другим домашним животным. Целью настоящего исследования была идентификация факторов, влияющих на инфицирование вирусом бешенства популяции людей в сельском сообществе Восточной Танзании.

Методы: Используя схему перекрестного исследования, были проведены полевые выезды с целью сбора информации о знаниях сельских жителей и об их практических действиях, связанных с борьбой с бешенством собак и кампанией вакцинации собак.

Результаты: Всего было опрошено 248 лиц в округе Килоса Танзании. Почти две трети из них (61,3%) имели начальное школьное образование. Большинство (91,1%) респондентов были осведомлены о том, что бешенство приобретается от укусов собак, и 66,9% знали о клинических признаках бешенства у животных. Однако, очень немногие (17,7%) знали о клинических признаках бешенства у человека. Лишь 20,4% респондентов знали о том, как бороться с бешенством у собак, и 71% не знали о кампании по вакцинации собак. Среднее число собак, содержащихся в домохозяйствах, составляло 4 ± 3,3; 70,0% респондентов имели от одной до пяти собак, 28,3% имели от шести до 10 собак и 1,6% имели по 16-20 собак. Собаки использовались, в основном, для охраны скота и имущества и для охоты. Около 59,7% респондентов указали, что бешенство является проблемой для общественного здоровья. В изучаемой области наблюдался низкий охват вакцинацией, а предыдущие кампаний массовой вакцинации охватили лишь 24,4% популяции собак. Представляется, что собаки имеют ограниченную ценность в исследуемом сообществе. Кроме того, не было надлежащим образом оборудованных мест для выброса отходов и дикие псовые и кошачьи часто заходили в деревни, подбирая кухонные остатки.

Заключение: Несмотря на то, что сообщества в округе Килоса имели знания о бешенстве у собак, они не осознавали связь заболевания с общественным здоровьем, что, таким образом, привело к недостаточному реагированию во время кампаний массовой вакцинации собак. Учреждение хорошо скоординированной программы борьбы с бешенством, организация стратегических кампаний просвещения по вопросам

общественного здоровья и систем пассивного мониторинга за людьми и домашними и дикими животными должны рассматриваться как стратегии для борьбы с бешенством и его искоренения.

Translated from English version into Russian by Alexander Somin, through

¿Por qué la rabia canina es endémica en el distrito de Kilosa, Tanzania? Lecciones aprendidas y soluciones que se deben tomar

M. J. Kipanyula

Resumen

Antecedentes: Los perros domésticos son los objetos principales para controlar la rabia ya que constituyen el principal reservorio de transmisión del virus de la rabia a los seres humanos y a otros animales domésticos. Este estudio tenía como objetivo identificar los factores que contribuyen a la rabia que infecta la población humana en una comunidad rural situada al este de Tanzania.

Métodos: Usando un diseño de estudio transversal, se realizaron visitas de campo para recopilar información sobre los conocimientos de los aldeanos, las prácticas asociadas a las campañas de vacunación de perros y el control de la rabia canina.

Resultados: Un total de 248 personas fueron entrevistadas en el distrito de Kilosa, Tanzania. Casi dos terceras partes (61.3%) tenía una educación primaria. La mayoría (91.1%) de los encuestados estaban conscientes de que la rabia se transmite a través de mordeduras de perro y 66.9 % conocían los signos clínicos de rabia en un animal. Sin embargo, muy pocos (17,7%), conocían los signos clínicos de rabia en los seres humanos. Sólo 20.4 % de los encuestados sabía cómo se controla la rabia en los perros y 71% no estaban al tanto de las campañas de vacunación de perro. La mediana de perros por hogar fue de 4 ± 3.3; 70.0 % de los encuestados tenía entre uno a cinco perros, 28.3 %tenía entre seis a 10 perros, y 1.6% tenía entre 16 a 20 perros. Los perros servían sobre todo para cuidar ganado, propiedades o para cazar. Un 59,7 % de los encuestados indicaron que la rabia era un problema de salud pública. Se observó una cobertura baja de vacunación en el área de estudio, las campañas masivas de vacunación sólo cubren el 24.4 % de la población canina. Los perros parecen tener un valor limitado en la comunidad estudiada. Además, no hay instalaciones de eliminación de residuos apropiadas y a menudo los felinos y caninos silvestres vienen a las aldeas para alimentarse con las sobras.

Conclusión: Aunque las comunidades en el distrito de Kilosa conocían la rabia canina, no estaban consciente de las implicaciones en la salud pública de esta enfermedad, lo cual se refleja en las respuestas negativas durante las campañas de vacunación masiva de perros. Se debe considerar como estrategia para controlar y erradicar la rabia: el establecimiento de coordinación de un programa de control de la rabia, la estrategia de campañas de concientización de salud pública y un sistema de vigilancia activa y pasiva para los seres humanos, los animales domésticos y silvestres.

Translated from English version into Spanish by Karina Atencio, through

TRANSLATORS
WITHOUT BORDERS
